# Molecular Epidemiology of *Blastomyces gilchristii* Clusters, Minnesota, USA

**DOI:** 10.3201/eid2809.220392

**Published:** 2022-09

**Authors:** Ujwal R. Bagal, Malia Ireland, Annastasia Gross, Jill Fischer, Meghan Bentz, Elizabeth L. Berkow, Anastasia P. Litvintseva, Nancy A. Chow

**Affiliations:** Centers for Disease Control and Prevention, Atlanta, Georgia, USA (U.R. Bagal, M. Bentz, E.L. Berkow, A.P. Litvintseva, N.A. Chow);; Minnesota Department of Health, St. Paul, Minnesota, USA (M. Ireland, A. Gross, J. Fischer)

**Keywords:** blastomycosis, fungi, respiratory infections, *Blastomyces gilchristii*, whole-genome sequencing, Minnesota, United States

## Abstract

We characterized 2 clusters of blastomycosis cases in Minnesota, USA, using whole-genome sequencing and single-nucleotide polymorphism analyses. *Blastomyces gilchristii* was confirmed as the cause of infection. Genomic analyses corresponded with epidemiologic findings for cases of *B. gilchristii* infections, demonstrating the utility of genomic methods for future blastomycosis outbreak investigations.

Three pathogenic *Blastomyces* species, *B. dermatitidis*, *B. gilchristii*, *and B. helicus*, have been identified in North America. In the United States, *B. dermatitidis* has been found throughout areas surrounding the Great Lakes, the Ohio and Mississippi River valleys, and the St. Lawrence River (*1*). In contrast, *B. gilchristii* has a smaller geographic range in Canada and the northern United States (*2*), and *B. helicus* has been found in the northwestern United States (*3*). No differences in clinical manifestations have been reported among these *Blastomyces* species.

In the United States, previous case reports have linked blastomycosis infections to outdoor activities, especially those involving moist soil and proximity to waterways (*4*,*5*). One of the largest reported outbreaks of blastomycosis occurred in 2015 among persons who had recreated along the Little Wolf River in Wisconsin (*6*). In Minnesota, blastomycosis is a reportable disease; epidemiologists at the Minnesota Department of Health (MDH) routinely collect demographic and clinical information for blastomycosis cases and attempt interviews to characterize illness and exposure history. The MDH Public Health Laboratory provides fungal identification services and stores isolates submitted by clinical laboratories.

Although whole-genome sequencing has been used to investigate outbreaks involving various fungal pathogens, such as *Candida auris* and *Coccidioides* spp. (*7*,*8*), this molecular technology has not been used to investigate *Blastomyces* spp. outbreaks in the United States. We performed whole-genome sequencing to determine the genetic diversity and phylogenetic relationships of 2 familial clusters of *B. gilchristii* infections identified in Minnesota.

In August 2020, five cases of blastomycosis were identified as cluster A, which comprised a family of 2 White Hispanic parents and 3 children (Table). Four of the 5 patients were hospitalized, of which 3 had sputum cultures that were positive for *Blastomyces* sp. All 5 patients recovered from illness. The mother reported that the family had visited rivers in St. Croix County, Wisconsin, numerous times during the summer. No other likely exposure locations or activities were reported. 

**Table:** Demographic and clinical data used for molecular epidemiology of 2 *Blastomyces gilchristii* clusters, Minnesota, USA*

Sample no.	Age, y/sex	Race/ethnicity	Cluster	Family relationship	Diagnosis location	Exposure location†	Clinical specimen	Specimen collection date
B19405	15/F	White Hispanic	A	Sister	MN	WI	Sputum	2020 Aug 23
B19406	27/M	White Hispanic	A	Brother	MN	WI	Sputum	2020 Aug 19
B19407	3/F	White non-Hispanic	B	Daughter	MN	MN	Bronchial washing	2020 Jul 25
B19408	38/M	White non-Hispanic	B	Father	MN	MN	Subcutaneous abscess	2014 Dec 16

*Data for 2 isolates per cluster that underwent whole-genome sequencing and single-nucleotide polymorphism analyses. †Likely exposure location on the basis of interviews with family members.

In addition, 2 cases of blastomycosis were identified in White non-Hispanic sisters. Only 1 sister was hospitalized and had a positive culture for *Blastomyces* sp. from a bronchoalveolar lavage specimen. MDH learned that their father had blastomycosis in 2014, which was attributed to *B. dermatitidis* (*9*). The 2 patients with isolates (1 sister and the father) were classified as cluster B (Table). The family owned a cabin in Hubbard County, Minnesota, which is highly endemic for blastomycosis and was likely the exposure location for the three cases. All 3 patients recovered from illness.

*Blastomyces* identification is routinely performed by MDH only at the genus level. Therefore, the Centers for Disease Control and Prevention (CDC) determined the species in 4 isolates from the 2 blastomycosis clusters and performed Illumina (https://www.illumina.com) short-read sequencing (National Center for Biotechnology Information BioProject accession no. PRJNA786864). To investigate genetic diversity between strains, we performed whole-genome single-nucleotide polymorphism (SNP) analysis using the MycoSNP version 0.19 analytical workflow (https://github.com/CDCgov/mycosnp). We used publicly available sequences from *B. dermatitidis* isolates (NCBI run nos. SRR11849827, SRR11849828, SRR11849829) for comparison and genome assembly data for *B. gilchristii* strain SLH14081 from GenBank (accession no. GCA_000003855.2) as a reference. We constructed a neighbor-joining tree showing SNP differences and maximum-likelihood tree showing bootstrap values using MEGA software version 7.0, (https://www.megasoftware.net) and FastTree 2 (*10*). 

All the isolates were *B. gilchristii* rather than *B. dermatitidis*. Phylogenetic tree analysis showed *B. dermatitidis* and *B. gilchristii* grouped into distinct clades, which were separated by 52,431 SNPs (Figure). Sequences from all 4 *B. gilchristii* isolates clustered with the reference genome SLH14081 and were separated by a minimum of 11,695 SNPs. Each familial cluster formed a subclade within the *B. gilchristii* clade; the subclades were separated by 5,214 SNPs. In cluster A, where all family members were infected at the same time and location, we found 63 SNPs separated the 2 cases. In cluster B, where exposures occurred in the same location but infections were 6 years apart, the cases differed by 120 SNPs (Figure).

**Figure:**
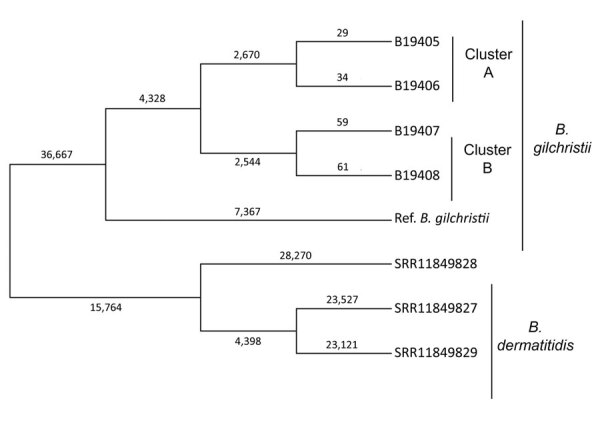
Genetic relationships and molecular epidemiology of *Blastomyces gilchristii* clusters, Minnesota, USA. We performed whole-genome sequencing of isolates from 4 patients in Minnesota who had *Blastomyces gilchristii* infections and compared the sequences with 3 publicly available *B. dermatitidis* isolates (National Center for Biotechnology Information run nos. SRR11849827, SRR11849828, SRR11849829). We analyzed single-nucleotide polymorphisms (SNPs) using the MycoSNP version 0.19 analytical workflow (https://github.com/CDCgov/mycosnp). We used the genome assembly data for *B. gilchristii* strain SLH14081 from GenBank (accession no. GCA_000003855.2) as a reference. Neighbor-joining tree shows the genetic relationships between cluster A and B, which each comprised isolates from 2 patients, the *B. gilchristii* reference strain, and *B. dermatitidis* isolates. Numbers represent the SNPs for each strain. Ref., reference.

Both *B. dermatitidis* and *B. gilchristii* have been reported in Minnesota (*2*). We used whole-genome sequencing and SNP analysis to evaluate clusters of blastomycosis infections caused by *B. gilchristii* in Minnesota. The genomic data showed that cases within cluster A or B were closely related genetically, whereas clusters A and B were genetically distinct. *B. gilchristii* is likely responsible for a higher proportion of blastomycosis clusters than is currently known. Therefore, pairing genomic data with clinical information and geographic location can be used to monitor blastomycosis infections and determine whether they are clusters, outbreaks, or sporadic occurrences. Our findings demonstrate the utility of genomic analyses for investigating blastomycosis outbreaks, determining genetic diversity of *B. dermatitidis* and *B. gilchristii*, and identifying common sources of environmental exposures among cases. 

## References

[1] Furcolow ML, Busey JF, Menges RW, Chick EW. Prevalence and incidence studies of human and canine blastomycosis. II. Yearly incidence studies in three selected states, 1960-1967. Am J Epidemiol. 1970;92:121–31. 10.1093/oxfordjournals.aje.a121184 5464317

[2] McTaggart LR, Brown EM, Richardson SE. Phylogeographic analysis of *Blastomyces dermatitidis* and *Blastomyces gilchristii* reveals an association with North American freshwater drainage basins. PLoS One. 2016;11:e0159396. 10.1371/journal.pone.0159396 27428521 PMC4948877

[3] Schwartz IS, Wiederhold NP, Hanson KE, Patterson TF, Sigler L. *Blastomyces helicus*, a new dimorphic fungus causing fatal pulmonary and systemic disease in humans and animals in western Canada and the United States. Clin Infect Dis. 2019;68:188–95. 10.1093/cid/ciy483 29878145 PMC6321858

[4] Klein BS, Vergeront JM, DiSalvo AF, Kaufman L, Davis JP. Two outbreaks of blastomycosis along rivers in Wisconsin. Isolation of *Blastomyces dermatitidis* from riverbank soil and evidence of its transmission along waterways. Am Rev Respir Dis. 1987;136:1333–8. 10.1164/ajrccm/136.6.1333 3688635

[5] Reed KD, Meece JK, Archer JR, Peterson AT. Ecologic niche modeling of *Blastomyces dermatitidis* in Wisconsin. PLoS One. 2008;3:e2034. 10.1371/journal.pone.0002034 18446224 PMC2323575

[6] Thompson K, Sterkel AK, Brooks EG. Blastomycosis in Wisconsin: Beyond the Outbreaks. Acad Forensic Pathol. 2017;7:119–29. 10.23907/2017.014 31239964 PMC6474482

[7] Chow NA, Gade L, Tsay SV, Forsberg K, Greenko JA, Southwick KL, et al.; US Candida auris Investigation Team. Multiple introductions and subsequent transmission of multidrug-resistant Candida auris in the USA: a molecular epidemiological survey. Lancet Infect Dis. 2018;18:1377–84. 10.1016/S1473-3099(18)30597-8 30293877 PMC6556114

[8] Oltean HN, Etienne KA, Roe CC, Gade L, McCotter OZ, Engelthaler DM, et al. Utility of whole-genome sequencing to ascertain locally acquired cases of coccidioidomycosis, Washington, USA. Emerg Infect Dis. 2019;25:501–6. 10.3201/eid2503.181155 30789132 PMC6390764

[9] Kaka AS, Sarosi GA. Disseminated Blastomycosis. N Engl J Med. 2017;376:e9. 10.1056/NEJMicm1606811 28177861

[10] Price MN, Dehal PS, Arkin AP. FastTree 2—approximately maximum-likelihood trees for large alignments. PLoS One. 2010;5:e9490. 10.1371/journal.pone.0009490 20224823 PMC2835736

